# Brain PET and SPECT imaging and quantification: a survey of the current status in the UK

**DOI:** 10.1097/MNM.0000000000001736

**Published:** 2023-07-18

**Authors:** Sofia K. Michopoulou, John C. Dickson, Glen G. Gardner, Thomas R. Gee, Andrew J. Fenwick, Timothy Melhuish, Clare A. Monaghan, Neil O’Brien, Angus M.J. Prosser, Catherine J. Scott, Roger T. Staff, Jonathan Taylor

**Affiliations:** a Clinical and Experimental Sciences, Faculty of Medicine, University of Southampton; b Imaging Physics, University Hospital Southampton, Southampton; c Institute of Nuclear Medicine, University College London Hospitals, London; d Department of Nuclear Medicine, NHS Tayside, Dundee; e National Physical Laboratory, Teddington; f Imaging Physics, NHS Grampian, Aberdeen; g Nuclear Medicine & 3DLab, Sheffield Teaching Hospitals, Sheffield, UK

**Keywords:** brain, dementia, epilepsy, neuroimaging, Parkinson disease, PET, quantification, SPECT

## Abstract

**Objectives:**

With disease-modifying therapies in development for neurological disorders, quantitative brain imaging techniques become increasingly relevant for objective early diagnosis and assessment of response to treatment. The aim of this study was to evaluate the use of Brain SPECT and PET scans in the UK and explore drivers and barriers to using quantitative analysis through an online survey.

**Methods:**

A web-based survey with 27 questions was used to capture a snapshot of brain imaging in the UK. The survey included multiple-choice questions assessing the availability and use of quantification for DaTscan, Perfusion SPECT, FDG PET and Amyloid PET. The survey results were reviewed and interpreted by a panel of imaging experts.

**Results:**

Forty-six unique responses were collected and analysed, with 84% of responses from brain imaging sites. Within these sites, 88% perform DaTscan, 50% Perfusion SPECT, 48% FDG PET, and 33% Amyloid PET, while a few sites use other PET tracers. Quantitative Brain analysis is used in 86% of sites performing DaTscans, 40% for Perfusion SPECT, 63% for FDG PET and 42% for Amyloid PET. Commercial tools are used more frequently than in-house software.

**Conclusion:**

The survey showed variations across the UK, with high availability of DaTscan imaging and quantification and lower availability of other SPECT and PET scans. The main drivers for quantification were improved reporting confidence and diagnostic accuracy, while the main barriers were a perception of a need for an appropriate database of healthy controls and a lack of training, time, and software availability.

## Introduction

Brain SPECT and PET imaging provide valuable information in diagnosing neurological conditions, including Parkinson’s, epilepsy, and dementia [1–5]. In dementia diagnosis, PET and SPECT can pick up early functional changes before these become visible in structural imaging techniques like CT and MRI [6]. Consequently, the UK National Institute for Care and Excellence (NICE) guidance for dementia recommends using PET and SPECT for patients with cognitive complaints with a negative or inconclusive structural brain scan [7]. Additionally, NICE recommends dopamine transporter imaging (DaTscan) for people with tremor if essential tremor cannot be clinically differentiated from Parkinsonism [8].

Quantitative analysis of brain scans may improve clinical diagnosis and is recommended by international guidance for certain indications [9,10]. Several studies have compared visual to quantitative reporting for brain SPECT and PET. Table 1 compares diagnostic accuracy for visual reporting alone versus combined visual and quantitative reporting for DaTscan, Perfusion SPECT, FDG PET and Amyloid PET. Quantitative reporting, including visual review, provides a clear advantage to visual reporting alone. In the case of DaTscan, the use of quantification results in a reduction of variability between reporting clinicians [11,12]. In Perfusion SPECT, FDG PET and Amyloid PET quantification, there are clear improvements in diagnostic accuracy, with quantification providing a clear cut-off that supports better differentiation between normal and pathological scans [9,19,20].

**Table 1:** Representative examples of studies comparing the performance of visual versus combined visual and quantitative reporting for the four types of scans reviewed in this survey

Scan type	Authors	Visual reporting	Visual and quantitative reporting
DaTscan	Soderlund, 2013 [11]	Interobserver agreement 0.8	Interobserver agreement 0.95
DaTscan	Booij, 2017 [12]	Accuracy 79% Reporting confidence 4.25	Accuracy 86% Reporting confidence 4.37
Perfusion SPECT	Frisoni, 2013 [13]	Sensitivity 68% Specificity 84%	Sensitivity 81% Specificity 83%
Perfusion SPECT	Imabayashi, 2004 [14]	Accuracy 74%	Accuracy 86%
FDG PET	Perani, 2014 [15]	Sensitivity 78% Specificity 50%	Sensitivity 96% Specificity 84%
FDG PET	Foster, 2007 [16]	Sensitivity 96% Specificity 59%	Sensitivity 98% Specificity 73%
Amyloid PET	Barthel, 2011 [17]	Sensitivity 80% Specificity 91%	Sensitivity 85% Specificity 91%
Amyloid PET	Camus, 2021 [18]	Sensitivity 85% Specificity 38%	Sensitivity 92% Specificity 91%

With an ageing population and disease-modifying therapies becoming available for neurological disorders, quantitative brain imaging techniques have become increasingly relevant for early diagnosis and assessment of treatment response. In the case of Amyloid PET, for example, where amyloid targeting treatments like lecanemab, aducanumab and donanemab have recently become available, quantification may offer the improved sensitivity required to support earlier identification of patients who may benefit from treatment, supporting stratification. Additionally, quantifying amyloid burden on longitudinal scans enables monitoring of treatment response.

Despite the evidence for the clinical and potential operational gains of using quantitative software in brain tomography, it remains unclear if its use has translated into broad practice. Anecdotally, our impression is that adoption is variable, and the reasons for non-adoption differ.

In this study, we present the results of a web-based survey which provides a snapshot of PET and SPECT brain imaging and quantification across the UK, comment on current practice, identify barriers and consider opportunities for future development.

## Methods

A web-based survey with 27 questions was developed. Most questions were multiple-choice with additional fields for adding comments. The survey was distributed through the Medical Physics and Engineering JISC mail base, the Institute of Physics and Engineering in Medicine (IPEM) communities of interest and the heads of the nuclear medicine physics group email list.

An overview of the survey results was shared back through the same platforms, and a panel of nuclear medicine physics experts was invited to a focus group meeting to discuss and interpret the survey findings. The survey form is provided in supplementary materials, with a short description of key questions and the rationale for asking those outlined here.

### Availability of brain imaging

*Q1. Do you perform Brain SPECT or PET at your site?*

*Q2. If no, why not?*

These questions were aimed to identify availability of brain scans and to identify the reasons why these are not offered at some sites.

### Imaging type availability, indication, quantification, volume of scans for common scan types

The following sets of questions were repeated for DaTscan, Perfusion SPECT, FDG PET and Amyloid PET. Here X denotes the scan type.

*Q3. Do you perform X at your centre?*

When the answer was ‘No’ the survey moved to the next scan type. When ‘Yes’ additional questions were asked about the use of this scan type at each centre.

*Q4. Do you perform X for Clinical or Research purposes?*

This was a multiple-choice question with answers 1. Clinical, 2. Research, 3. Clinical and Research, to help establish the current use for the different scan types.

*Q5. For which indications? (please select all that apply*)

This was a multiple-choice question, outlining as options the most common indications (i.e. for DaTscan there were options for Parkinson’s and Dementia with Lewy Bodies) and also providing the option of ‘Other’ encouraging participants to provide additional indications relevant to their sites.

*Q6. Do you use software for quantitative analysis?*

This was a multiple-choice question offering the following options 1. No quantitative analysis, 2. Quantitative analysis using in-house developed software, 3. Quantitative analysis using commercially available software, 4. Other. This question helped evaluate the availability of quantification and the split between using in-house developed versus commercial for each type of scan.

*Q7. Would you describe the volume of X scans at your site as 1. Low (<2 per month), 2. Medium (2–10 per month), 3. High (>10 per month).*

This helped assess the frequency of scans and evaluate associations between scan volumes and availability of quantification at each site.

### Availability of other PET or SPECT brain scans

*Q23. Do you perform any other scans for neurological indications (select all that apply*)

This was a multiple-choice question offering the options of 1. Cardiac MIBG, 2. Tau PET, 3. DOPA PET, 4. TSPO PET, and 5. Other, where participants were encouraged to outline less common brain scans that are available at their centres.

### Drivers and barriers for quantitative analysis

*Q24. What are the main drivers for performing quantitative analysis of brain scans at your centre?*

The options for this question were 1. Improved Reporting Confidence, 2. Improved Diagnostic Accuracy, 3. Alignment with Guidelines, and 4. Other.

*Q25. What are the main barriers for performing quantitative analysis of brain scans at your centre?*

The options for this question were 1. Not required, not used by reporting clinician, 2. Lack of database of healthy controls, 3. Lack of dedicated software, 4. Lack of expertise, training, 5. Lack of time, 6. No barriers and 7. Other.

*Q26. Do you have any further comments/ thoughts you’d like to add?*

This is intended to capture additional information not covered in the multiple-choice questions and clarifications on previous answers.

*Q27. Which site is your response for?*

This is to help confirm the responses are from UK sites and to remove duplicates. Site-identifying information was removed for data processing and presentation of results.

## Results

### Overview of responses

**Fig. 1:**
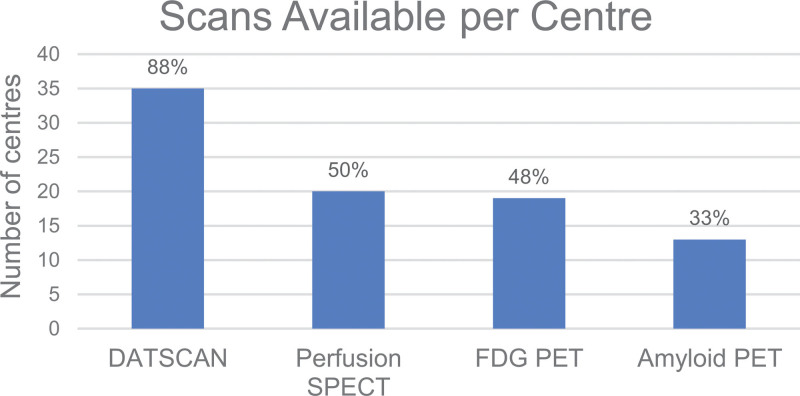
Scan type availability across the responding centres.

**Fig. 2:**
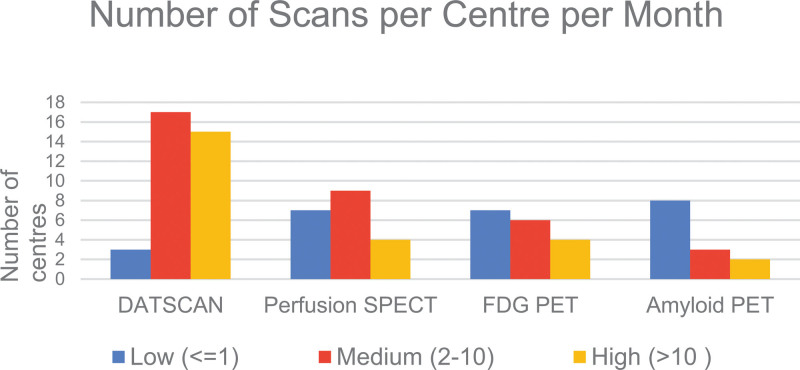
Intended scan use for clinical or research purposes by scan type.

**Fig. 3:**
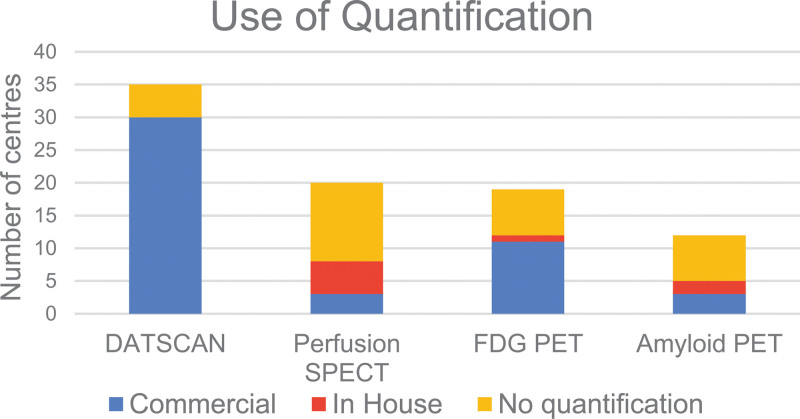
Availability and use of quantification by scan type, with the specification for commercial or in-house software solutions.

**Fig. 4:**
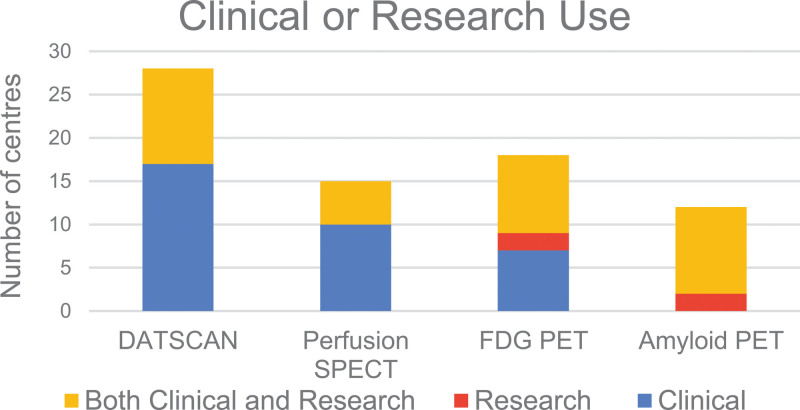
Scan volume per month, expressed as low, medium or high, per scan type.

A total of 52 responses were received from a mixture of district hospitals and university hospitals across the UK. Six responses were identified as duplicates based on the name of the institution, and they were removed by only retaining the latest duplicate submissions based on the timestamp of the survey response. The results of unique responses from 46 nuclear medicine departments across the UK are presented here. These represent 17% of 269 nuclear medicine departments in the UK, as recorded in the 2021 British Nuclear Medicine Society (BNMS) survey. The following sections will summarise the results of the survey answers, while Figs. 1–4 provide an overview of these results for the four types of imaging included in the survey.

### Availability of brain imaging

Out of 46 responders, six centres do not offer brain imaging, while 40 offer at least one type of brain imaging. The limited number of responses from centres that do not perform brain imaging is to be expected and likely represents a selection bias as the survey was focused on brain imaging and quantification. Centres not performing these scans would be less likely to respond to the survey. The reasons for not offering brain imaging were (1) inappropriate equipment, (2) lack of patients, (3) being an oncology-specific centre, (4) referring patients to a nearby centre, and (5) using a different imaging modality as local consultants have low confidence in reporting SPECT.

### DaTscan

Out of 40 responders, 35 (88%) provided DaTscan imaging. The primary indication is Parkinson’s disease, with scans performed in 35/35 centres, followed by Lewy Body Dementia, where scans are offered in 32/35 centres. In terms of DaTscan quantification, this is available in 30/35 (86%) centres. All 30 centres currently use the commercially available software. Regarding the volume of scans, 52% have high, 40% have medium, and 8% have a low number of scans.

### Perfusion SPECT

Out of 40 responders, 20 (50%) provide Perfusion SPECT imaging. The primary indication is Dementia, with scans performed in 15/20 centres, followed by epilepsy, where scans are offered in 9/20 centres. In terms of Perfusion SPECT quantification, this is available in 8/20 (40%) centres. From these, three centres use commercially available software, and five centres use in-house developed solutions. Regarding the volume of scans, 20% have high, 45% have medium, and 35% have a low number of scans.

### FDG PET

Out of 40 responders, 19 (47.5%) provide FDG PET imaging. The primary indication is Dementia, with scans performed in 14/19 centres, followed by epilepsy, where scans are offered in 9/19 centres. Regarding FDG PET quantification, this is available in 12/19 (63%) centres. Eleven centres use commercially available software, and 1 uses in-house developed software. Regarding the volume of scans, 22% have high, 33% have medium, and 45% have a low number of scans.

### Amyloid PET

Of 40 responders, 13 (32.5%) provide Amyloid PET imaging. The primary indication is Alzheimer’s Disease, with scans performed in 13/13 centres. Eighty-three percent of centres perform Amyloid PET for both clinical and research purposes, while 17% of centres perform this scan for research purposes only. In terms of Amyloid PET quantification, this is available in 5/12 (42%) responding centres. From these, two centres use commercially available software and three use in-house developed software. Regarding the volume of scans, 15% have high, 23% have medium, and 62% have a low number of scans.

### Other scans for neurological indications

Regarding other scans performed for neurological indications, eleven centres perform Cardiac MIBG scans, six centres perform Tau PET, four centres perform Dopa PET, two centres perform TSPO PET, while brain receptor PET and methionine PET are performed in one centre each.

### Drivers for quantification

The main drivers for quantification were the following: improved reporting confidence in 32/34 (94%), improved diagnostic accuracy in 29/34 (85%), and alignment with guidelines in 14/34 (41%), while 2 (6%) centres reported that quantification is required for research purposes including drug development.

### Barriers to quantification

The main barriers to quantification were the following: lack of a database of healthy controls in 9/36 (25%), lack of time in 9/36 (25%), quantification not required or not used by the reporting clinician in 8/36 (22%), lack of dedicated software in 6/36 (17%), lack of expertise and training in 6/36 (17%). 13/36 (36%) reported no barriers to quantification at their centres.

## Discussion

The response to the survey was reasonable, with 17% of UK Nuclear Medicine sites responding. Broadly, the results confirmed our subjective impression that the clinical and operational gains of quantitative brain analysis are not being exploited.

### Availability of brain PET and SPECT imaging

Only 13% of sites reported they do not offer brain imaging. From centres who responded that they do not perform brain imaging using SPECT, one of the reasons was the low confidence in reporting, which is an area where the use of quantification is of value [12].

DaTscan is the most widely available scan (88% of responders), used for clinical and, in some cases, research applications. It is a well-established imaging modality that NICE recommends should be available to specialists with expertise in its use and interpretation for Parkinson’s and for differential diagnosis of Dementia with Lewy bodies [7,8].

Perfusion SPECT and FDG PET are available in almost equal measure (~50% of responders), with a slightly higher volume of scans for SPECT. They are both used primarily for clinical purposes, with some use for research. NICE only recommends SPECT when PET is not available [7]. Responders commented on the lack of access to PET in their sites and the limited capacity of PET scanners, as scanning slots were reserved for imaging in oncology. For these reasons, it is expected that perfusion SPECT will continue being used in the foreseeable future until there is an improvement in PET access for brain imaging [1].

Amyloid PET is available in only a third of centres and is used for research with some clinical applications. The use of Amyloid PET in clinical trials is primarily for developing and evaluating new drugs for Alzheimer’s disease. Currently, this scan is not funded by NICE for routine clinical use. However, if new disease-modifying drugs get approval in the UK, the use of Amyloid PET is expected to increase as it is a prerequisite for patient stratification [21].

In terms of other scan types being available, 28% of centres perform cardiac MIBG scans, and 15% of centres perform tau PET. In contrast, other brain PET radiopharmaceuticals are used in a smaller number of centres. Further information is required to understand the use and availability of quantification for these scans, as the survey did not include further questions on these.

### Availability of quantification, drivers and barriers

**Fig. 5:**
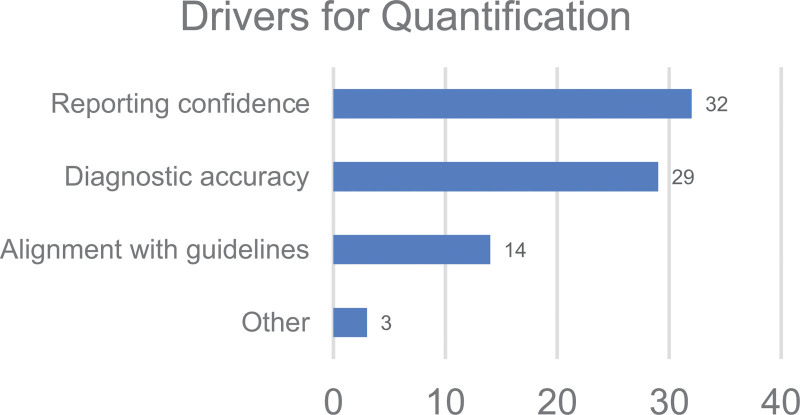
Key drivers for quantification across the responding centres.

As shown in Fig. 5, the key drivers for quantification were reported to be improving reporting confidence and increasing diagnostic accuracy. This is well supported by the published evidence, summarised in Table 1, that demonstrates the substantial benefits of quantification as part of clinical reporting across the four scan types reviewed in this survey. Despite the benefits being clear, the use of quantification is limited for certain scan types.

**Fig. 6:**
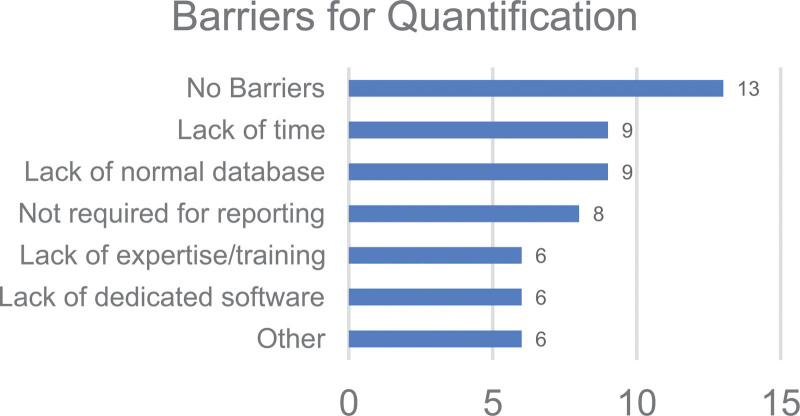
Key barriers for quantification across the responding centres.

As shown in Fig. 6, various barriers to quantification were reported, including the availability of software, the lack of time and availability of appropriate normal databases. The fourth most common barrier identified by 22% of responders is that quantification is not required or not used by the reporting clinician. Given the clear benefits of quantification for brain PET and SPECT, clinicians should be provided with training to support them in integrating quantification into clinical reporting. The BNMS could lead this effort by providing training courses for its members and engaging with the Royal College of Radiology to update its curriculum to include quantification in the core training for nuclear medicine clinicians and neuroradiologists.

Taking the perceived barriers to quantification in turn, the lack of software availability tops the list. Even though all major vendors supply a version of quantitative software, this comes with additional costs, often limiting on-site availability. Quantification is often considered an optional extra, as it is not included as a requirement on clinical guidelines. However, as the evidence base on the added value of quantification has grown over the last decade, quantitative analysis has now been included as a recommendation in the latest guidelines, which is helpful to departments trying to justify the added costs of software purchase [10,22]. The next barrier identified is lack of time which has two components, (1) the time required for setting up, evaluating the software and training the team on its use, and (2) the time needed for executing the software for each patient scan. The time required for setup, evaluation and training could be reduced if direct support from the software manufacturer was provided, as has been the case for DaTscan for UK-based users of the DATQuant software. The additional time for processing each patient scan can be better managed when this is performed in real-time through integration with the standard clinical post-processing workflows on the acquisition workstation or on PACS. However, legacy software is sometimes available in a separate processing environment requiring additional data transfer and time. The third barrier was in relation to the availability of appropriate normal databases, which includes considerations about the number of healthy controls included, the acquisition and reconstruction parameters used, and the age range covered in the database, as there are different age requirements, for example, for dementia and epilepsy indications. Pre-comparison registration and smoothing can mitigate many differences between the database scans and scans acquired at each centre. Attention should be paid to using equivalent collimators to those used in the database scans or providing appropriate conversions to support the portability of normal databases [23]. Scanner calibration may be required in certain cases [24]. Age-related changes are substantial and should be considered carefully [25,26]. More information on normal databases is provided along with other scan-specific drivers and barriers in the following subsections.

Whilst the benefits of quantification have been demonstrated, it is likely that there is variability in quantitative PET and SPECT accuracy between centres which should be identified. A national audit could provide useful information in this regard and help to guide improvements in methodology. Assessment of uncertainty is also important to enable meaningful comparison of data and could be used to guide clinicians when reporting.

#### DaTscan

Quantification is widely used in DaTscan (86%), based on commercial software solutions. There is strong evidence that DaTscan quantification improves reporting interobserver agreement and reporting confidence [11] and enables readers with limited experience to have a diagnostic accuracy equivalent to that of experienced readers [12]. The survey results showed that the top driver for using quantification is to improve diagnostic confidence. A further reason for the widespread use of DaTscan quantification is the availability of software coupled with scanner-agnostic normal databases [27]. For example, the European multicentre database of healthy controls for [123I]FP-CIT SPECT (ENC-DAT) database includes healthy controls across the lifespan, has well characterised imaging protocols and is supported by evidence that, when accounting for reconstruction parameters, differences between scanners have limited impact on quantification, enabling portability to different scanners [24,25]. In this survey, all sites reported they use commercial quantitation tools for DaTscan, and a wide range of such platforms are available, including Scenium, BRASS, MIM Neurology and DATQuant. The radiopharmaceutical manufacturer provides the latter, and also supports scanner protocol setup using a striatal phantom, facilitating UK-based users in setting up DaTscan imaging and quantification.

#### Perfusion SPECT

Survey results showed that the use of quantification is very limited for perfusion SPECT scans (40%). There is strong evidence that quantification increases the diagnostic accuracy of perfusion SPECT [20,28]. Frisoni *et al*. reported a sensitivity improvement of 13% when using quantification compared to visual reporting [13]. Imabayashi *et al*. reported a 12% improvement in quantitative accuracy when using quantification [14]. Semi-quantitative SPECT has also been shown by Prosser *et al*. to be valued by referring clinicians and to improve clinician diagnostic confidence [29]. The use of quantification is crucial for optimising the diagnostic performance of perfusion SPECT and is recommended by the EANM guidelines [9,30]. However, responders highlighted the lack of quantification software as a key barrier to implementing quantification. Perfusion SPECT quantification was reported to rely more frequently on in-house developed software. Commercial quantification software can be an expensive add on and is more likely to be made accessible when included as a requirement with scanner procurement. Scanner-agnostic normal databases, such as those used in DaTscan, may help unlock perfusion SPECT quantification. The portability of normal databases is achievable through harmonising imaging and reconstruction protocols and performing phantom-based checks [31–33]. Adopting phantom-based checks similar to those available for DaTscan imaging in the UK should help increase the use of quantification. There may be a reluctance to put time and funding towards implementing quantification and database harmonisation for perfusion SPECT for those sites that intend to replace this with FDG PET in future. However, considering the wide availability of SPECT coupled with the limited availability of PET for brain studies, it is anticipated that SPECT will continue to be used in the foreseeable future [1]. The substantial benefits in diagnostic performance would justify future efforts to enable wider use of quantification in perfusion SPECT.

#### FDG PET

FDG PET is more widely quantified (62%) than perfusion SPECT, primarily using commercial software but lagging behind DaTscan. It is well-established that FDG PET quantification enhances diagnostic accuracy and increases confidence in dementia diagnosis [10]. Foster *et al*. found that quantification increases the specificity of FDG PET by 14% compared to visual reporting alone [F18]. Kono *et al*. showed that PET quantification improves the differentiation between dementia subtypes, such as Dementia with Lewy Bodies versus Alzheimer’s disease [34]. Multiple commercial software solutions with in-built normal databases are available for FDG PET quantification. However, the cost of commercial software may be challenging to justify for centres performing a limited number of scans. Some PET centres operating under the UK national PET contract have access to a centrally procured software licence, which may present a more cost-effective approach and support the standardisation of quantification procedures. Protocol harmonisation is available from the EARL FDG brain accreditation scheme based on the Hoffman phantom [35]. Furthermore, the Quantitative Imaging Biomarkers Alliance has developed and made openly available Digitally Reference Objects, which manufacturers could use to provide an objective evaluation of software performance which could facilitate the selection of the most appropriate software solution for a particular setting [36].

#### Amyloid PET

Amyloid PET sees limited quantification, with only 42% of scanning sites quantifying scans. Since many of the Amyloid PET scans are performed as part of clinical trials and submitted for central reporting, on-site quantification may not currently be relevant to local sites for those studies. However, clinical Amyloid PET imaging and quantification are expected as new anti-amyloid treatments require amyloid positivity for patient stratification [21]. Amyloid PET quantification improves sensitivity compared to visual reporting alone [17,18]. A quantitative cut-off could enable earlier identification of candidates for treatment and aid in patient stratification to different treatment options. The scanner accreditation pathways and protocol harmonisation procedures for anti-amyloid drug trials provide a good basis for setting up quantitative imaging pathways for Amyloid PET. Software procurement and training would be the next hurdle toward increasing the use of Amyloid PET quantification. Although not widely used, quantitative software is available for these scans. A large validation that evaluated 15 different software methods for amyloid quantification showed comparable results between different processing tools and concluded that amyloid quantification methods could complement the visual analysis and support early identification of Amyloid deposition [37]. Should Amyloid PET become commonplace in the diagnostic pathway, support for its use would be required.

### Limitations

Although many individual responses (46) were collected, these represent only 17% of UK-based imaging centres. There are very few responses (6) from centres not performing brain imaging, and hence more information is required to understand better the barriers to performing SPECT and PET brain imaging.

Due to the survey being circulated through medical physics portals and the expert panel being primarily clinical scientists, the results collected, and associated discussion mostly represent the opinions of this professional group, who are routinely responsible for implementing imaging protocols and quantification. Hence, the survey needs more input from other stakeholders. Further work should be done to identify drivers and barriers across the different healthcare professions.

The survey was focussed on the UK, aiming to provide a local snapshot of the current use of imaging and quantification. Expanding this work to other countries would help further identify barriers and drivers for brain PET and SPECT imaging and quantification for different healthcare systems.

### Conclusion and recommendations

The survey showed variations across the UK, with high availability of DaTscan imaging and quantification and lower availability of perfusion SPECT and FDG and Amyloid PET scans. The key drivers identified are supported by extensive literature highlighting substantial benefits for diagnostic accuracy and reporting confidence. Quantification should be considered an essential part of brain imaging to help optimise diagnostic performance. For this purpose, the key barriers to quantification identified in this survey should be addressed. Capital purchase of brain quantification software as part of new equipment procurement could improve accessibility. Learning from DaTscan, scanner agnostic normal databases could enable wider implementation, for example, in Perfusion SPECT and Amyloid PET. Protocol harmonisation, following, for example, the EARL methodology, would improve consistency between centres. Assessment of uncertainty in quantitative brain imaging would allow better comparison between centres. Establishing a national audit would help to identify the current ‘state-of-the-art’ and guide future research. Dedicated training for quantification as part of clinical reporting is urgently required to support clinicians in harvesting the benefits of quantification when interpreting perfusion SPECT, FDG PET and Amyloid PET.

## Acknowledgements

The authors would like to thank all responders to the survey. Sofia Michopoulou and Catherine Scott are funded through Integrated Clinical Academic Lectureship by Health Education England (HEE)/NIHR (NIHR301287 and NIHR302139). The views expressed in this publication are those of the authors and not necessarily those of the NIHR or the UK Department of Health and Social Care. Andrew Fenwick is employed by the National Physical Laboratory, his contribution was funded by the UK Government’s Department for Science, Innovation & Technology (DSIT) through the UK’s National Measurement System programmes.

### Conflicts of interest

Prof. John Dickson is a Consultant for Clario. For the remaining authors, there are no conflicts of interest.
